# Editorial: Exploring future thinking processes in intertemporal choice and delay discounting

**DOI:** 10.3389/fpsyg.2026.1939554

**Published:** 2026-09-17

**Authors:** Evgeniya Lukinova, Valérie Godefroy, Cristiano Valerio dos Santos

**Affiliations:** 1 N/LAB, University of Nottingham, Jubilee Campus, Nottingham, United Kingdom; 2 Université Bourgogne Europe, Institut Agro, CNRS, INRAe, UMR CSGA, DOCC lab, Dijon, France; 3 Centro de Estudios e Investigación en Comportamiento, University of Guadalajara, Guadalajara, Mexico

**Keywords:** cognitive processes, delay discounting, future thinking processes, intertemporal choice, self-control

This Research Topic was launched to advance understanding of the psychological, biological, and contextual mechanisms that shape how people evaluate future outcomes and make choices involving trade-offs between immediate and delayed rewards.

## Environmental and demographic determinants of time preferences

Current research in cognitive science increasingly demonstrates that psychological states induced by the environment play a fundamental role in decision-making. Rather than being fixed internal traits, our preferences for time and risk are dynamic responses to the stability, predictability, and resource levels of our surroundings. The first two manuscripts in this Research Topic provide compelling evidence for this view, exploring how socioeconomic context and environmental volatility warp the internal psychological mechanisms, such as time perception and emotional resilience, that govern our choices.

The first manuscript, authored by Mitra and Srivastava, investigates how “precarity” (unpredictable environmental events like income shocks or expense hikes) affects intertemporal choice. The researchers move beyond standard economic models that assume fixed discount rates, instead proposing that environmental instability shifts preferences by distorting perceived time. Using a computer-based farming simulator, participants navigated stable (high profitability) and volatile (high loss) conditions while choosing between a non-risky short-term crop (rice), a risky short-term crop (apple), and a non-risky long-term crop (teak). The second manuscript, by Wan et al., explores how age and income interact to influence decision-making regarding both delayed and probabilistic (risky) rewards. By examining a diverse sample of adults aged 20 to 80, the researchers tested the “buffering hypothesis,” which posits that age-related increases in emotional stability can protect individuals against the psychological stressors of their environment.

Together, these manuscripts show that human decision-making is inseparable from the environment. Mitra and Srivastava demonstrate that environmental volatility may dilate the psychological experience of time, making the short-term plans appear more valuable. Wan et al. complement this by showing that environmental scarcity creates a state of psychological distress that older adults may be better equipped to handle due to increased emotional stability. Both studies underscore that to understand why people choose the way they do, we must first understand the psychological climate created by the world they inhabit.

## Biological and neural mechanisms of intertemporal choice

Recent research increasingly suggests that delay discounting is shaped by the interaction of multiple neurobiological and cognitive processes that influence how future rewards are represented, anticipated, and evaluated. For example, Zhao et al. (2025) linked the association between reward sensitivity and delay discounting to functional connectivity within valuation-related neural networks, while a large genome-wide association study identified 11 loci associated with delay discounting and demonstrated extensive genetic overlap with psychiatric, cognitive, physical health, and neuroimaging phenotypes (Thorpe et al., 2026). Consistent with the objectives of this Research Topic, three contributions illustrate how endocrine, neurophysiological, and behavioral markers can advance our understanding of delay discounting by highlighting complementary mechanisms through which biological state variables, anticipatory processing of future outcomes, and reward sensitivity influence choice.

In their research article, Lukinova and Erlich examined whether biological factors are associated with delay discounting across timescales using complementary measures of stress, including self-reported stress, salivary cortisol, and hair cortisol. Their findings demonstrate that endogenous cortisol fluctuations are associated with changes in willingness to wait over seconds, but not days, highlighting the dynamic influence of stress-related endocrine processes on time preferences.

Extending the investigation to neurophysiological mechanisms, Zakirov and Sysoeva examined EEG correlates of impulsive choice during an experiential delay discounting task with delays varying over seconds. Their brief research report revealed that greater self-control is associated with reduced alpha-band activity during reward anticipation, suggesting that sustained attentional focus during waiting may support tolerance of delayed rewards.

Research article by Davidovich et al. broadened the methodological scope of this Research Topic by pairing delay discounting measures with lottery choice and a biologically grounded measure of reward sensitivity. Sweet-likers were more risk-averse and preferred immediate rewards when contrasted with delayed alternatives, indicating that the relationship between reward sensitivity and impulsivity may reflect preferences for certainty rather than greater sensitivity to reward magnitude alone.

## Delay discounting in clinical and occupational contexts

In this Research Topic, two reviews of literature examined delay discounting in specific populations, among patients with neurocognitive and mental disorders as well as among workers. Studying delay discounting in patients with transdiagnostic approaches constitutes an opportunity to identify shared cognitive and neural mechanisms of delay discounting. Moreover, focusing on specific populations provides insight into the potential concrete uses that delay discounting tasks may have within a particular context.

The review by Dubois et al. among patients with Alzheimer's disease, depression or PTSD, has collected evidence suggesting a shared central mechanism underlying their steeper delay discounting rates (or more impulsive choices). Impaired episodic future thinking (EFT)—the difficulty to imagine events that may happen in the future—systematically co-occurs with discounting modifications across these three conditions and potentially other conditions. In the three conditions, high delay discounting is associated with reduced specificity in EFT, reduced memory specificity as well as neural network disruptions involving hippocampus and ventromedial prefrontal cortex. Thus, the difficulty to project in the past and future seems to jeopardize the ability to make choices with future-oriented goals. Interventions targeting an improvement of EFT could be beneficial to restore more typical discounting but successful interventions in these populations showing impaired EFT might create specific challenges.

In the population of workers and within organizations, the review of literature by Escobar and Vega-Valero shows several examples of the relevance of using delay discounting in work contexts. Some studies for instance used framing effects (that is narratives or hypothetical decision scenarios) in association with the delay discounting task to investigate how different contexts may impact impulsive decision-making at work. Other studies focused on the potential role of individual differences in delay discounting in shaping career paths, job selection or other work-related decisions such as timing of retirement.

The seven contributions collected in this Research Topic reflect the diversity of contemporary research on intertemporal choice, examining how future-oriented processes operate across different populations, reward types, and temporal horizons, while drawing on interdisciplinary approaches.

## References

[B1] Thorpe H. H. Cupertino R. B. Pakala S. R. Fontanillas P. Jennings M. V. Yang J. . (2026). Genome-wide association study of delay discounting identifies 11 loci and reveals transdiagnostic associations across mental and physical health. Mol. Psychiatry 31, 2081–2093. doi: 10.1038/s41380-025-03356-8 41286433 PMC12999501

[B2] Zhao H. Wei Z. Feng T. Feng P. (2025). The neural substrates responsible for reward sensitivity association with delay discounting: left dorsal anterior cingulate cortex connectivity with right precuneus. Brain Struct. Funct. 230, 1–17. doi: 10.1007/s00429-025-03028-x 41091286

